# Reconstruction Accuracy Assessment of Surface and Underwater 3D Motion Analysis: A New Approach

**DOI:** 10.1155/2015/269264

**Published:** 2015-06-14

**Authors:** Kelly de Jesus, Karla de Jesus, Pedro Figueiredo, João Paulo Vilas-Boas, Ricardo Jorge Fernandes, Leandro José Machado

**Affiliations:** ^1^Centre of Research, Education, Innovation and Intervention in Sport (CIFI2D), Faculty of Sport, University of Porto (FADE-UP), 4200-450 Porto, Portugal; ^2^Porto Biomechanics Laboratory (LABIOMEP), University of Porto (UP), 4200-450 Porto, Portugal; ^3^School of Physical Education, Federal University of Rio Grande do Sul (UFRGS), 90040-060 Porto Alegre, RS, Brazil

## Abstract

This study assessed accuracy of surface and underwater 3D reconstruction of a calibration volume with and without homography. A calibration volume (6000 × 2000 × 2500 mm) with 236 markers (64 above and 88 underwater control points—with 8 common points at water surface—and 92 validation points) was positioned on a 25 m swimming pool and recorded with two surface and four underwater cameras. Planar homography estimation for each calibration plane was computed to perform image rectification. Direct linear transformation algorithm for 3D reconstruction was applied, using 1600000 different combinations of 32 and 44 points out of the 64 and 88 control points for surface and underwater markers (resp.). Root Mean Square (RMS) error with homography of control and validations points was lower than without it for surface and underwater cameras (*P* ≤ 0.03). With homography, RMS errors of control and validation points were similar between surface and underwater cameras (*P* ≥ 0.47). Without homography, RMS error of control points was greater for underwater than surface cameras (*P* ≤ 0.04) and the opposite was observed for validation points (*P* ≤ 0.04). It is recommended that future studies using 3D reconstruction should include homography to improve swimming movement analysis accuracy.

## 1. Introduction

The application of a multidigital camera set-up for three-dimensional (3D) analysis is frequently implemented in controlled indoor or laboratory settings [1, 2]. However, its use outdoors or in constrained environments for specific sport applications is very limited [3]. Furthermore, in specific underwater conditions there are a number of technical issues (e.g., camera arrangement, calibration and protocol methodology, and motion data collection) that lead to a preference of a two-dimensional (2D) data collection (on one side of the body, assuming the existence of a bilateral symmetry [4]). This 2D approach might be less complex to use in traditional aquatic settings, but it implies a higher occurrence of errors by disregarding the multiplanar nature of the swimmers' movement characteristics [5].

Complementarily, manual tracking is the most used method to detect and follow the trajectory of body anatomical landmarks and calibration points (often attached to a custom static support recorded by each video camera field of view) during underwater movement quantitative analysis (e.g., [6]). With this process, the coordinates of the calibration points are registered in each camera 2D field of view, allowing a 3D movement reconstruction through the use of the direct linear transformation (DLT) algorithm [7]. Previous findings revealed that the increase in number (e.g., from 8 to 20–24 [4, 5, 7]) and wider distribution [7, 8] of the control points as well as the decrease in the calibration volume size [9, 10] had improved the 3D reconstruction accuracy for surface and/or underwater cameras. Nevertheless, large calibration volumes are needed in swimming analysis since they minimize data extrapolation beyond the calibrated space, increasing further measurements accuracy [11]. Moreover studies have often reported larger errors for underwater camera views and have justified them through light refraction (water has higher refraction index than air) and consequently image deformation.

In addition, for a more accurate 3D reconstruction, the displacement of each pixel across the images (induced by camera, scene position, and/or independent object-motion) should also be controlled [12–15]. For this purpose, homography is considered as a key step to obtain mappings between scene images, since computing homographies is faster and less erroneous than the motion process structure. This is justified by the fact that the homography parameters are determined by few corresponding points [13, 14], being typically estimated between images by finding feature correspondence. To the best of our knowledge, no research in swimming kinematics has considered the homography as a transformation method for 3D image rectification; we aimed to compare the 3D reconstruction accuracy in a large and static calibration volume (for surface and underwater digital video) using different calibration point sequences. The homography technique was applied to correct control points in each camera field of view and compared with the nonhomography implementation. Following Nejadasl and Lindenbergh [13], it was hypothesised that implementing homography technology would improve 3D reconstruction accuracy. Moreover, it is expected that, using homography or not, underwater cameras would display greater 3D reconstruction errors than surface cameras.

## 2. Material and Methods

### 2.1. Static 3D Calibration Volume

**Figure 1:**
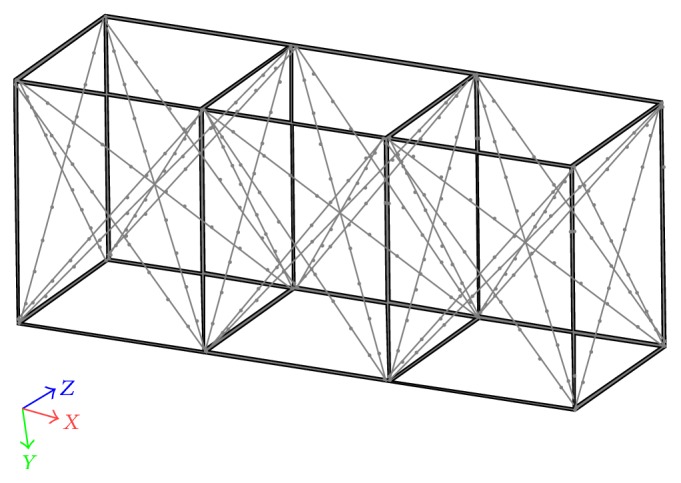
The rectangular prism used as the static calibration volume.

A 3D calibration volume was designed using the software Solid Works 2013 (3D CAD Premium, Dassault Systèmes SolidWorks Corporation, USA; Figure 1), being based on rigid structures used in previous swimming related studies [4, 5, 9]. Afterwards, it was built using a computer numerical control machine and was comprised of three blocks, each one with the following dimensions: (i) 2000 mm length, 2500 mm height, and 2000 mm width. These parts were framed and joined to form a rectangular prism of 6000 × 2500 × 2000 mm^3^ (with a total calibration space of 30 × 10^9^ mm^3^), enabling the record of at least two complete consecutive swimming cycles. The 3D coordinate accuracy of the calibration volume was 1.2 mm for horizontal (*x*) and vertical (*y*) and 1.4 mm for lateral axes (*z*).

The calibration volume structure was manufactured in anodised aluminium with 25 mm diameter, selected on the basis of its high flexural stiffness relative to its weight, allowing reduced distortions due to frequent research use or/and to the swimming pool environment [16]. Stainless steel cables (5 mm) were used to triangulate each frame part, ensuring that the adjoining sides of the frame followed orthogonality. Two hundred and thirty-six black tape markers (15 mm width each) were attached with 250 mm separation on the aluminium tubes in the *x*-, *y*-, and *z*-axes. A laser device was used to improve the accuracy of markers placing (Nano, Wicked Lasers©, Hong Kong). The 3D coordinate's accuracy of the markers was 0.5 mm for *x* and *y* and 0.9 mm for *z*.

### 2.2. Data Collection

**Figure 2:**
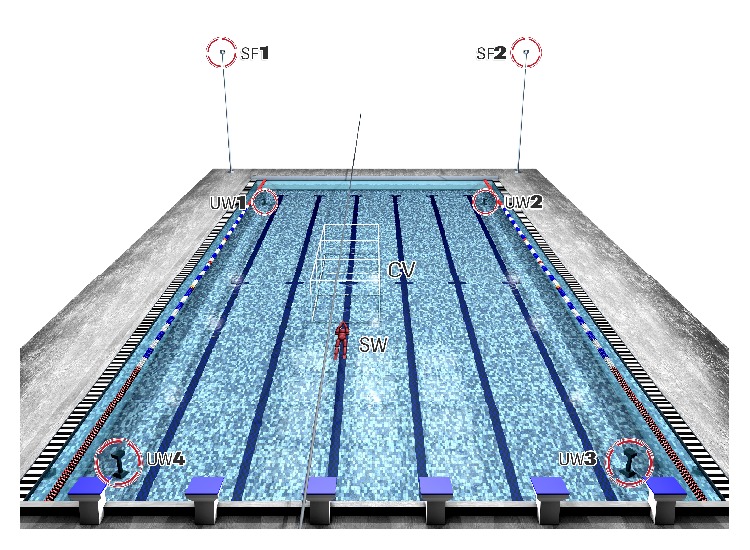
Experimental 3D camera set-up. Cameras UW1, UW2, UW3, and UW4: 1st, 2nd, 3rd and 4th underwater cameras. Cameras SF1 and SF2: 1st and 2nd surface cameras. Calibration volume: CV. Swimmer: SW.

The 236 calibration points distribution in the calibration volume was registered simultaneously by four underwater and two surface water stationary video cameras (HDR CX160E, Sony Electronics Inc., Tokyo, Japan) recording at 50 Hz. The calibration volume was positioned in the centre of a 25 m swimming pool (1900 mm depth) and its longitudinal axis was aligned with the lateral wall of the swimming pool.  Figure 2 shows the calibration volume and the 3D camera set-up: the surface and underwater cameras were placed at an equal distance from the respective centre, forming an angle of 100° between the axes of the two surface water cameras while the angle established by the underwater cameras varied between 75 and 110° [5].

The surface cameras were positioned in tripods (Hamma Ltd., Hampshire, UK) at 3.5 m (height) and the underwater cameras were maintained in a waterproof housing (SPK-HCH, Sony Electronics Inc., Tokyo, Japan) and fixed on tripods at 1.0 to 1.5 m (depth). A LED system visible in each video camera field of view was used for image synchronisation.

### 2.3. Data Analysis

The 236 points on the calibration volume with known coordinates were manually digitised (Matlab version R2012a, Mathworks, Inc.) to obtain their (*u*, *v*) coordinates and the DLT method was applied for 3D reconstruction according to [17]

(1) u=L1x+L2y+L3z+L4L9x+L10y+L11z+1,v=L5x+L6y+L7z+L8L9x+L10y+L11z+1.

To evaluate the quality of manual digitisation procedure, a specific routine in the Matlab software was developed to identify the difference between real and estimated coordinate values. The routine consisted in classifying the digitised points into large, medium, and small errors, being (i) large error, represented by red colour (error > 25 mm), (ii) medium error, represented by orange colour (15 mm < error < 25 mm), and (iii) small error, represented by green and blue colours (error ≤ 15 mm). After this analysis, depending on the results obtained, the points were redigitised until optimal value achievement. A limit of 25 mm for the difference between the real and estimated coordinates was imposed for each camera view and several points have shown errors in the range of 25 and 33 mm, which was a hint to the use of manual homography transformation to assign the real coordinates to each projected point and to avoid possible mistakes.

Under linear projection, the mapping from a pixel (*u*, *v*) to a control point (*x*, *y*, 0) on the calibration plane (*z* = 0) is encapsulated by homography matrix *H* as

(2) $$\begin{array}{c} \left(\begin{array}{c} x\\ y\\ 1 \end{array}\right)=H_{3\times 3}\left(\begin{array}{c} u\\ v\\ 1 \end{array}\right)=\left(\begin{array}{ccc} h_{11} & h_{12} & h_{13}\\ h_{21} & h_{22} & h_{23}\\ h_{31} & h_{32} & h_{33} \end{array}\right)\cdot \left(\begin{array}{c} u\\ v\\ 1 \end{array}\right). \end{array}$$

Given at least four point correspondences (*u*_*i*_, *v*_*i*_)→(*x*_*i*_, *y*_*i*_, 0), the homography can be estimated by solving the overdetermined homogeneous linear system:

(3) u1v11000−x1u1−x1v1−x1000−u1−v1−1y1u1y1v1y1u2v21000−x2u2−x2v2−x2000−u2−v2−1y2u2y2v2y2u3v31000−x3u3−x3v3−x3000−u3−v3−1y3u3y3v3y3u4v41000−x4u4−x4v4−x4000−u4−v4−1y4u4y4v4y4·h11h12h13h21h22h23h31h32h33=0.

The point correspondences are derived from the manually digitised calibration points and their real coordinates. Once the homography is estimated, a projected feature point detected at pixel (*u*_*p*_, *v*_*p*_) can be associated to its world coordinates according to (2).

**Figure 3:**
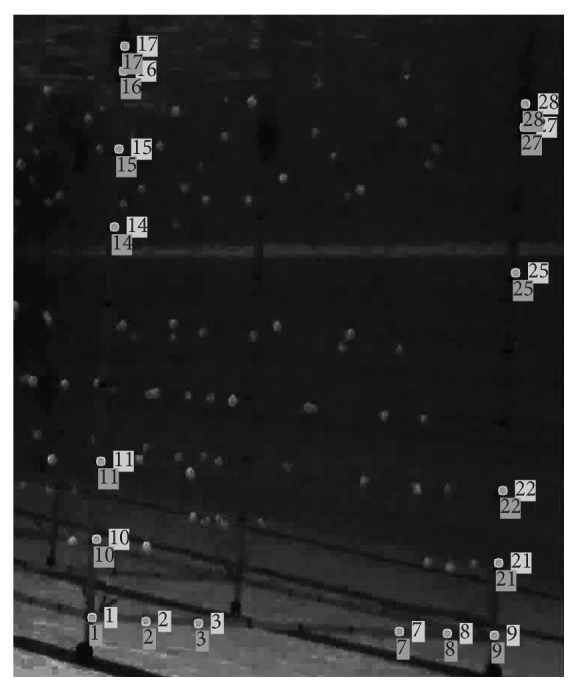
Visual comparison of 3D reconstruction for the homographic transformation of a calibration volume plane. Unnumbered squares: original points from digitising; cross on the unnumbered squares: point after homographic transformation.

During the manual homography analysis, the two camera sets (i.e., surface and underwater) were independent in between, as shown in Figure 3.

**Figure 4:**
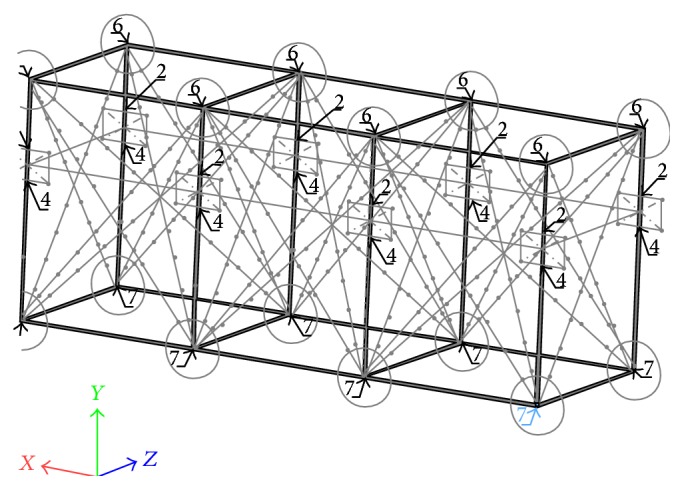
Location of the control points on the static calibration volume.

Of the 236 points on the calibration volume with known coordinates located at the horizontal and vertical rods making the calibration volume, a total of 64 surface and 88 underwater markers near the frame inner and outer corners and at the water line were selected to be the control points (circles and diamonds in Figure 4). The points at the water line were common to both surface and underwater control points. The remaining 92 points (38 surface and 54 underwater) were used as the validation points.

From each of those areas referred to above, points were systematically combined in sets of 3 per corner (whenever possible), resulting in sets of 40 and 48 calibration points for surface and underwater, respectively. From these calibration points, the DLT was performed and applied to the remaining control points and separately for the validation points.

Then, a new combination of calibration points from the control points was selected and a new DLT was again performed and applied to the remaining points. This systematic selection procedure resulted in over 1.5 million different combinations for the underwater control points and over 1000 combinations for the surface control points.

When the homography transformation was used to smooth the digitising errors, it was applied only to the control points and then the systematic selection procedure referred to above was used. To simplify, the homography transformation was applied to a plane defined by a given set of rods, for each camera separately, with the process being applied three times to each camera to account for the rods that are common to two planes. Validation points were also smoothed by the homography transformation; however these points will not be digitised in future uses of the calibration volume.

### 2.4. Accuracy

All reconstruction errors were calculated from the raw coordinate data, without any smoothing procedure [18], and determined by the Root Mean Square (RMS) error of the 92 validation points (for the total calibration volume), using the following equations:

(4) Xr=1N∑i=1Nxni−xi2,Yr=1N∑i=1Nyni−yi2,Zr=1N∑i=1Nzni−zi2,R=1N∑i=1Nxni−xi2+yni−yi2+zni−zi2,

where, *X*_*r*_, *Y*_*r*_, *Z*_*r*_, and *R* were the RMS errors for each axis and for the resultant error (resp.), *x*_*ni*_, *y*_*ni*_, and *z*_*ni*_ were the real coordinates, *x*_*i*_, *y*_*i*_, and *z*_*i*_ were the reconstructed coordinates, and *N* was the number of points used.

### 2.5. Statistical Analysis

Data are reported as mean and standard deviations (±SD). The normality distribution was checked and confirmed with Shapiro-Wilk's test. A two-way repeated measures ANOVA (homography × cameras) on control and validation points was performed after verifying sphericity (Mauchly's test). Pairwise multiple post hoc comparisons were conducted with Bonferroni's correction. The level of significance was set at *α* = 0.05 (2-tailed). All data were analyzed using the IBM Statistical Package for Social Sciences (SPSS) 20.0.

## 3. Results

**Figure 5:**
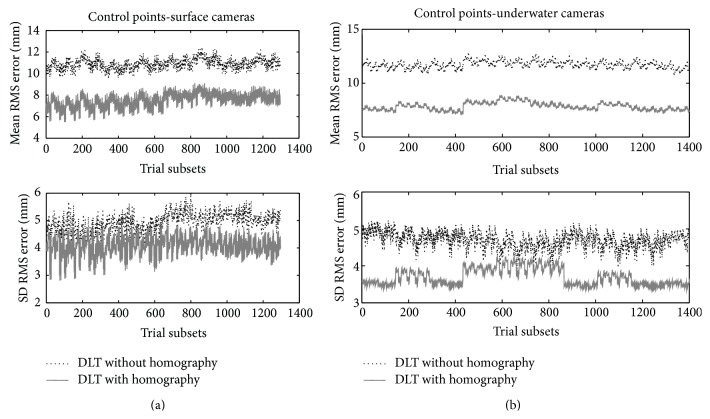
(a) RMS errors for the 3D reconstruction of surface cameras without (dotted line) and with homography (continuous grey line) transformation obtained from subsets of 40/64 control points positioned on the horizontal and vertical corner rods. Trial subsets in the *x*-axis represent the (arbitrary) ID of the simulation case with different subsets of control points. (b) RMS errors for the 3D reconstruction of underwater cameras without (dotted line) and with homography (continuous grey line) transformation obtained from subsets of 48/88 control points positioned on the horizontal and vertical corner rods. Trial subsets in the *x*-axis represent the (arbitrary) ID of the simulation case with different subsets of control points.

Figures 5(a) and 5(b) depicts the mean and SD values of the RMS errors (mm) for the 3D reconstruction of surface (over 1000 combinations of trial subsets of 40 points each from the set of 64 control points near the corners) and underwater (over 1600000 combinations of trial subsets of 48 points each from the set of 88 control points near the corners) cameras with and without homography transformation. Considering reconstruction through control point sets, homography use has revealed lower RMS errors for surface and underwater cameras rather than without it, being 7.3 ± 4.5 versus 10.5 ± 4.8 for surface (*P* < 0.01) and 7.7 ± 3.8 versus 12.1 ± 5.1 for underwater views (*P* < 0.01). Surface and underwater cameras have shown similar RMS error with homography (*P* = 0.47), although, without it, RMS error was greater for underwater than for surface cameras (*P* < 0.04).

**Figure 6:**
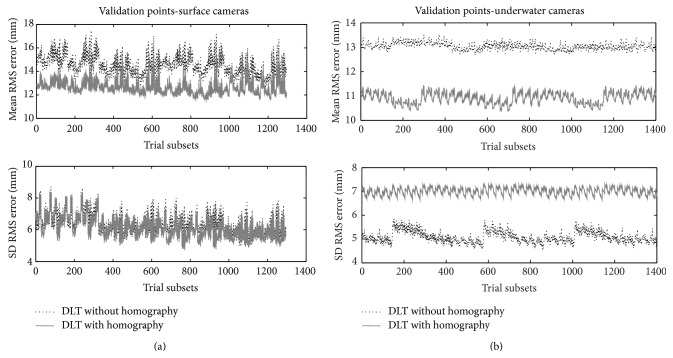
(a) RMS errors for 3D reconstruction with 92 validation points of the horizontal facets of surface (38 points) camera without (dotted line) and with homography (continuous grey line) transformation. Trial subsets in the *x*-axis represent the (arbitrary) ID of the simulation case with different subsets of control points. (b) RMS errors for 3D reconstruction with 92 validation points of the horizontal facets of underwater (54 points) camera without (dotted line) and with homography (continuous grey line) transformation. Trial subsets in the *x*-axis represent the (arbitrary) ID of the simulation case with different subsets of control points.

Figures 6(a) and 6(b) depict the mean and SD values of the RMS errors (mm) for 3D reconstruction of surface (38 validation points) and underwater (54 validation points) cameras with and without homography transformation. Regarding reconstruction through validation point sets, RMS error was lower with homography than without it for both cameras sets, being 12.1 ± 6.5 versus 15.9 ± 6.6 for surface (*P* < 0.01) and 10.8 ± 5.3 versus 13.3 ± 6.7 for underwater views (*P* < 0.03). Surface and underwater cameras evidenced similar RMS errors with homography (*P* = 0.49), but, without it, RMS reconstruction errors of surface were greater than underwater points (*P* < 0.04).

## 4. Discussion

The kinematic analysis in swimming imposes obstacles to data acquisition, particularly by the existence of errors associated with image distortion, digitalization, and 3D reconstruction [1, 19]. Thus, it is crucial to observe its influence on the final results, analysing validity, reliability, and accuracy [18]. To the best of our knowledge, the current study is the first that analysed the effects of homography and cameras positioning (surface/underwater) on 3D RMS reconstruction errors in swimming. Main findings were as follows: (1) using homography, RMS errors of control and validation points were smaller than without homography use and remained similar between surface and underwater cameras; (2) without homography, RMS errors of control points were greater for underwater rather than for surface cameras and, in opposition, RMS errors of validation points were greater for surface than for underwater cameras. These current findings partially confirm the already established hypotheses and suggested that homography method applied for surface and underwater cameras is suitable to minimize the error magnitude provided by large calibration volume dimensions.

Literature pointed out that the number of control points and its respective distribution on calibration volume are determinant for 3D reconstruction accuracy of surface and underwater cameras [4, 5, 7–9, 19]. In the current study, the numbers of control points distributed on the corners and facets for surface and underwater cameras were quite larger than those usually reported in swimming related studies [4–6, 9, 11]. The use of 8 to 30 control points distributed at the horizontal and vertical rods is often used for swimming 3D reconstruction with shorter calibration volume dimensions [4, 5] than those applied in the current study. Figure 4 revealed that the best set of control points was located on the corner and facets agreeing with previous study suggestions (e.g., [5]). As calibration volume size increases, it has been recommended to increase the number of control points with proper distribution to ensure accuracy augmentation [4, 7, 20]. Hence, researchers using static calibration structures with similar dimensions than those used in the current study should prioritize those criteria. Notwithstanding the number and location of control points as well as the calibration volume size relevance for better 3D reconstruction accuracy [7, 10], the effects of displacement of each pixel across the images induced by camera, scene position, and/or independent object-motion should also be considered in swimming analysis, since they have greatly affected reconstruction in other sport scenarios [13–15]. These drawbacks have been minimized through the use of different methods [21] being homography estimation well accepted as a key step to obtain mappings between scene images providing less erroneous 3D reconstruction [13].

In the light of those benefits provided by homography technique, its use was tested in swimming and has revealed a decrease in RMS errors of control and validation points for surface and underwater cameras, corroborating previous findings considering reconstruction from multiple perspective views [14, 15]. For example, Alvarez et al. [15] analysing competitive tennis observed a reduction of ≥ 10 mm on RMS error of control points when using homography estimation, which was higher than the current findings. In the present study, a reduction of 3 to 5 mm on RMS errors for both control and validation points in surface and underwater views was considered quite relevant due, especially for underwater cameras, to video recordings complexity in aquatic scenarios [19]. Differences between Alvarez et al. [15] study and the present study findings for surface RMS errors can be attributed to the greater incidence of light refraction and the smaller number of cameras used to record video images in swimming pool environment. Despite several previous findings considering underwater and surface 3D reconstruction analysis, the current study evidenced that swimming researchers should focus on homography implementation to test present results replication on their specific 3D cameras arrangements.

The control points and calibration volume sizes have not been an exclusive research topic in swimming 3D reconstruction studies, researchers also being interested in comparing RMS errors between underwater and surface cameras [4, 5, 9]. However, this problematic should not be considered as the major research concern, since specialized literature has evidenced greater underwater RMS errors rather than surface cameras prior to the 1990s (e.g., [22]). Researchers should focus on methods that allow minimizing errors from estimated to real coordinates of each camera, as homography has demonstrated. Implementation of homography has provided similar RMS errors for surface and underwater cameras, and these findings suggest for these sets of points that homography can be considered more advantageous for underwater reconstruction. Without homography, surface cameras reported lower RMS errors of control points than underwater cameras, as currently shown in literature [4, 5, 9]. These authors displayed RMS errors ranging from 4.06 to 6.16 mm for surface and 4.04 to 7.38 mm for underwater cameras, which were lower than the current results and that can be explained by differences in calibration volume sizes. Despite these differences, the large calibration volume used in the current study presented acceptable RMS errors of control points for surface and underwater cameras, avoiding the need of extrapolation beyond the calibrated space (e.g., [9]). The greater RMS error for surface than underwater cameras when considering validation points suggests that when homography is not used in large calibration volume dimensions, researchers should choose control instead of validation points for surface reconstruction.

## 5. Further Considerations

Notwithstanding the originality and relevance of the current data, some considerations should be taken into account. First, static calibration volumes remain by far the most widely used for swimming 3D reconstruction, although promising alternative calibration methods as chessboard and moving wand have shown interesting results [2, 3]. Nevertheless, these methods do not minimize extrapolation occurrence beyond the calibrated space, increasing measurements inaccuracy. The large calibration volume used in this study registered low and acceptable reconstruction accuracy errors to record at least two swimming cycles, but researchers are advised to take some cautions during video recording data collections. Second, manual digitisation process implies systematic and random errors [1]; however, in the current study they were kept in an acceptable level (≤8 mm) [10]. Third, the large number of control points used in the present study for surface and underwater reconstruction allowed obtaining low RMS error for a large calibration structure, although it is acknowledged that a minimum of six noncoplanar control points well distributed over the calibration volume can preserve adequate accuracy. Six control points recommendation can simplify digitisation process; however those points seem not enough to supply reliable reconstruction of large calibration volumes.

## 6. Conclusions

In the current study, the implementation of planar projective transformation through homography indicated that the RMS reconstruction errors of a set of 40/64 (surface) and 48/88 (underwater) control points positioned on the orthogonal corners and facets of a calibration volume with 6000 × 2500 × 2000 mm were similar and acceptable for surface and underwater views. Based on these findings, future studies using large calibration volumes able to record at least two cycles of a given swimming technique should consider homography transformation to smooth the digitised control points and improve the DLT reconstruction accuracy.
